# Commentary: enhancing methodological rigor in evaluating the efficacy of GnRH agonist in frozen-thawed embryo transfer

**DOI:** 10.5935/1518-0557.20250026

**Published:** 2025

**Authors:** Mehrab Neyazi, Rachana Mehta, Shubham Kumar, Ranjana Sah

**Affiliations:** 1 Afghanistan Center for Epidemiological Studies, Herat, Afghanistan; 2 Rachna International Institute of Research and Studies, Faridabad, Haryana 121004, India; 3 Saveetha University, Chennai, India; 4 Dr. D. Y. Patil Vidyapeeth (Deemed-to-be-University), Pimpri, Pune - 411018, Maharashtra, India

Dear Editor,

The recent systematic review and meta-analysis by Chienvichai *et al*. (2024), which evaluates the effect of a single-dose gonadotropin-releasing hormone agonist (GnRHa) on pregnancy outcomes in frozen-thawed embryo transfer (FET) cycles, contributes significantly to the field of reproductive medicine. The authors’ findings suggest that a single dose of GnRHa can potentially increase clinical pregnancy rates, particularly in natural cycle FETs. However, there are areas in the study’s methodology and analysis that could be enhanced to bolster the validity and applicability of the results.

The use of the GRADE (Grading of Recommendations Assessment, Development and Evaluation) approach could provide a more structured and transparent method for rating the quality of evidence and strength of recommendations. GRADE is particularly adept at addressing the variability in study quality and outcomes, which seems pertinent given the diverse set of RCTs included in the analysis (Dewidar *et al*., 2023; Pandey *et al*., 2024). By applying GRADE to each outcome rather than the studies as a whole, the authors could offer a clearer, outcome-specific assessment that supports more nuanced clinical decision-making.

A sensitivity analysis using the leave-one-out method could strengthen the study’s conclusions by demonstrating the robustness of the results. This approach involves sequentially removing each study to observe the impact on the overall effect size, thus identifying any particular study’s influence on the meta-analysis outcome(Higgins *et al*., 2019). Additionally, conducting sensitivity analyses based on the risk of bias categories would help clarify whether the results are disproportionately driven by studies with lower methodological rigor. This is crucial, as the meta-analysis results hinge on the assumption that all included studies are equally reliable.

While the study adeptly captures a broad range of outcomes, the lack of significant findings in secondary outcomes such as live birth rates and miscarriage rates raises questions about the clinical significance of using GnRHa in FET cycles. It would be beneficial for future research to explore the mechanisms through which GnRHa may influence early pregnancy outcomes without affecting later stages. This could involve more detailed investigations into endometrial receptivity and embryo-endometrium synchrony, potentially through advanced imaging techniques or molecular analyses. Moreover, the review could benefit from a more detailed discussion on the biological plausibility of GnRHa’s effects in different types of FET cycles. Understanding why GnRHa appears more effective in natural cycles could lead to tailored treatments that optimize individual patient protocols based on their specific reproductive health profiles.

This systematic review and meta-analysis provide intriguing insights into the potential benefits of GnRHa in enhancing clinical pregnancy rates in FET cycles, the application of GRADE, comprehensive sensitivity analyses, and a deeper exploration into the biological mechanisms at play would significantly enhance the reliability and impact of the findings. These enhancements would solidify the foundation of the review and guide future research directions in optimizing fertility treatments.
